# Editorial: Inflammation, stem cells and wound healing in skin aging

**DOI:** 10.3389/fcell.2022.1046022

**Published:** 2022-10-13

**Authors:** Mingxing Lei, Wen-Hui Lien, Ji Li

**Affiliations:** ^1^ 111 Project Laboratory of Biomechanics and Tissue Repair, Key Laboratory of Biorheological Science and Technology of the Ministry of Education, College of Bioengineering, Chongqing University, Chongqing, China; ^2^ de Duve Institute, Université catholique de Louvain, Brussels, Belgium; ^3^ Department of Dermatology, Xiangya Hospital, Central South University, Changsha, China; ^4^ Hunan Key Laboratory of Aging Biology, Xiangya Hospital, Central South University, Changsha, China; ^5^ National Clinical Research Center for Geriatric Disorders, Xiangya Hospital, Central South University, Changsha, China

**Keywords:** inflammation, stem cells, skin aging, wound healing, skin

## Introduction

Skin aging is the most recognizable consequence of senescence, mainly manifested as epidermal and dermal thinning, reduced elasticity, wrinkle formation, skin relaxation, abnormal skin pigmentation, wound healing disorders, hair graying, and pilosebaceous unit degeneration (Kohl et al., 2011; Kanaki et al., 2016; Gu et al., 2020). It is believed that the extrinsic skin aging primarily arises from UV-light exposure, whereas several factors are shown to induce intrinsic skin aging, including cellular senescence and the shortening of telomeres, mutations of mitochondrial DNA, oxidative stress, genetic mutations, and decreased levels of hormones, such as estrogen and progesterone (Kohl et al., 2011). Overall, skin aging is a highly complex but incompletely understood process, and despite great progress in recent years, many mysteries of aging mechanisms remain unsolved.

**FIGURE 1:**
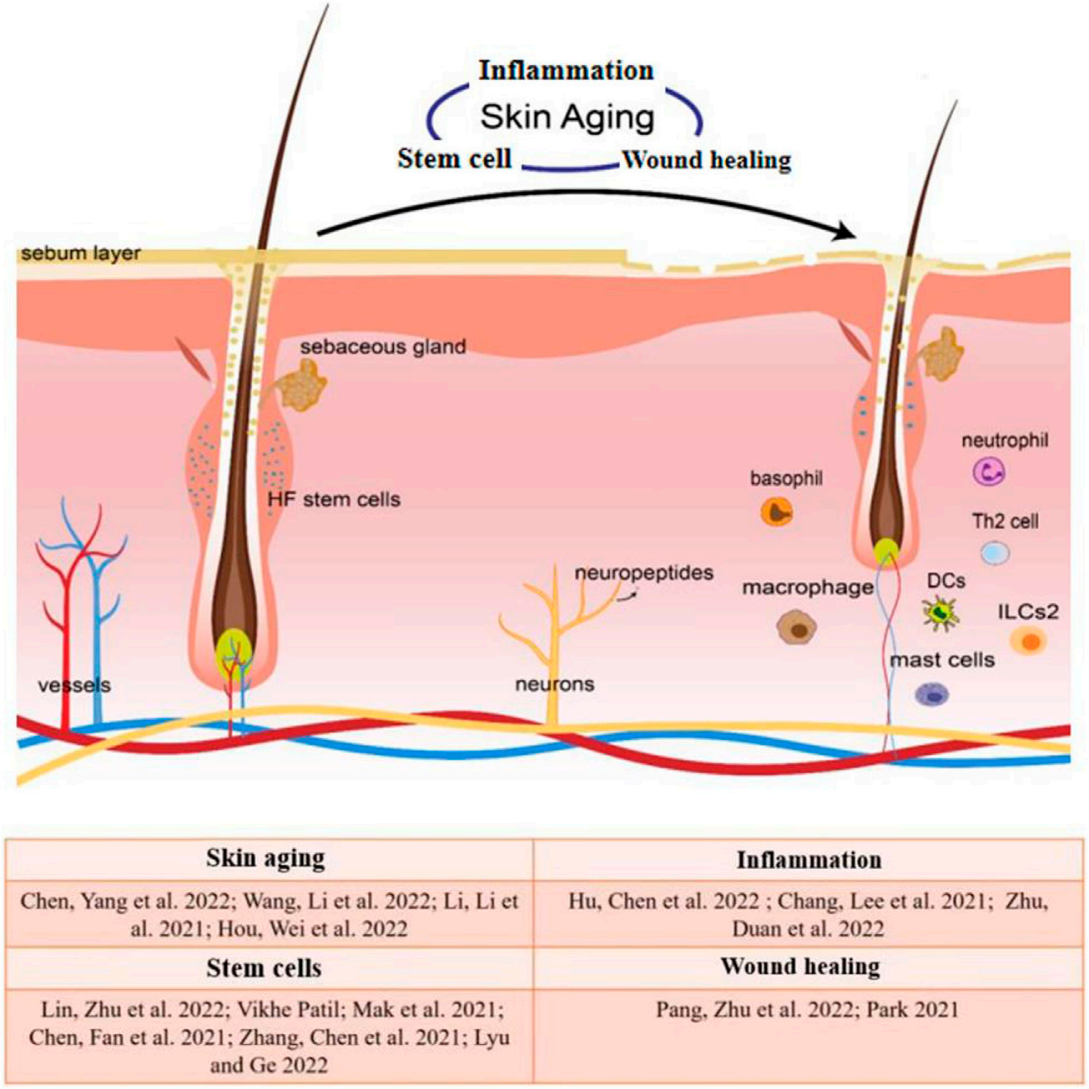
Inflammation, stem cells and wound healing in skin aging is characterized by thinned epidermal and dermal, damaged skin barrier, smaller hair follicles, fewer HF stem cells, and minimized DP. Skin aging leads to impaired stem cell activity, chronic inflammation, and compromised wound healing, and is also associated with neuroimmune and inflammatory disorders.

Skin is in direct contact with the environment outside the body and is stimulated by various external factors, such as UV radiations, microorganisms, etc. External and internal stimuli trigger the immune response of skin cells, causing a series of inflammatory reactions that induce inflammatory skin diseases, such as psoriasis, rosacea, atopic dermatitis, etc. Long-term chronic inflammation may induce skin cell senescence, and the compromised stem cell activity and wound responses are the consequence of skin aging (Figure 1). Therefore, a deep understanding of physiological regulation and pathological mechanisms of skin aging helps to advance the regenerative biology field and future clinical applications. Here we organize this Research Topic with a collection of original research and review articles that explore skin aging-related inflammation, stem cell activity, and wound healing. This collection aims to provide new insights into skin aging.

## Skin aging

Skin aging occurs under various circumstances, and light damage is considered to be the main exogenous cause. With the progress of aging, inflammation appears, and various profound structural and functional changes take place one after another (Hsu et al., 2014), all of which play either a positive or a negative regulatory role on aging in feedback. The two articles in this Research Topic discuss the latest viewpoints on skin aging in relation to light damage and skin structure. Light damage is an important risk factor for photoaging. Wang *et al.* found that HSP27 could play a protective role in UV irradiation-induced skin photoaging by stimulating autophagy and reducing reactive oxygen species (ROS) production; therefore, it may serve as a potential therapeutic target for photoaging (Wang et al.). Furthermore, the dermal extracellular matrix (ECM) constitutes the main framework of the dermis, and its composition changes greatly with skin aging. Li *et al.* analyzed the composition of dermal ECM in decellularized skin scaffolds of different age groups using a quantitative proteomics approach, and identified the regulatory pattern of ECM in the process of aging. Their results provide new clues for biomaterials that can be utilized in skin regeneration (Li et al.). Other than the above-mentioned aspects, many other factors are also involved in skin aging and anti-aging. Here we discuss three major factors that play crucial roles in skin aging, including inflammation, stem cells, and wound healing.

## Inflammation and skin aging

With aging, the whole body is progressing into a chronic low inflammatory state, which in turn accelerates aging in feedback by enhancing oxidative stress, DNA damage, and stem cell aging. The mini-review by Chen *et al.* summarized the relationship between type 2 inflammation and skin immunosenescence, and brought up the idea that skin inflammation and skin aging could regulate each other. They showed that chronic low levels of proinflammatory factors released by senescent cells could induce skin immunosenescence and inflammation, and suggested that it is promising to ameliorate inflammatory skin diseases by delaying skin immunosenescence (Chen et al.). The existing studies show that aging-related inflammation results in various diseases, such as hypertension (Liberale et al., 2022), diabetes (Bharath et al., 2020), and so on. In the skin, aging leads to functional damage of immune cells, fibroblasts, keratinocytes, etc., and then consequently causes chronic inflammation and immune diseases. Hu *et al.* revealed the dysregulation of invariant natural killer T (iNKT) cells in the pathogenesis of psoriasis and proposed suginumumab that targets a key factor of iNKT cells, IL-17, as a therapeutic drug (Hu et al.). Chang *et al.* reviewed the innate immune disorders in the pathogenesis and progression of vitiligo, including the early activation of NK cells, dendritic cells, and the involvement of various T cells, and proposed that immunomodulatory therapy is critical for vitiligo (Chang et al.). The senescence-associated secretory phenotype (SASP), involving high levels of inflammatory cytokines, chemokines, and matrix metalloproteinases, is considered to be the primary cause of the harmful effects of senescent cells. This strongly suggests the unavoidable effect of inflammation in inducing and promoting aging (Picardo et al., 2015). At present, it is generally believed that neuroimmune interactions play an increasingly important role in aging-related inflammation and are also the basis of the pathogenesis of these immune diseases. Zhu, Y. *et al.* described the mutual regulation of neural and immune systems in skin, and analyzed the neuroimmune mechanisms of various inflammatory skin diseases (Zhu et al.).

## Stem cells and skin aging

The decrease in the number and activity of stem cells is an inevitable change during skin aging, which leads to age-related alopecia and delayed wound healing. As the primary skin appendage, the hair follicle relies on hair follicle stem cells (HFSCs) to regenerate hair during skin homeostasis. HFSCs and hair follicle organoids are major models for studying hair-related diseases and skin aging (Lei and Chuong 2016; Lei et al., 2017). Six papers in this Research Topic highlighted the regulation and mechanisms in the development and degeneration of the pilosebaceous unit that consists of the hair follicle and sebaceous gland. Hou *et al.* systematically reviewed the signaling pathways and neuroendocrine changes during sebaceous gland differentiation and aging, and summarized the prevention and treatment measures against sebaceous gland aging (Hou et al.). Lin *et al.* delineated the morphological development, cycle, and molecular regulation of hair follicles (Lin et al.). Lyu *et al.* provided a comprehensive summary of the molecular mechanisms regulating hair follicle degeneration during aging. They outlined how nutrient sensing, metabolic reprogramming, altered mitochondrial activity, and epigenetic regulation affect hair regeneration, and affirmed the dominant role of the tissue microenvironment in regulating aging epithelial stem cell function (Lyu and Ge). Vikhe Patil *et al.* summarized the expression and function of peptidylarginine deiminases (PADIs), enzymes that convert amino acid arginine to citrulline, in the hair follicle stem cell lineage and inflammatory alopecia, and provided a comprehensive perspective on how citrullination modulates hair follicle regeneration and contributes to inflammatory alopecia (Patil et al.). Chen *et al.* revealed a new function of dermal white adipose tissue (dWAT) in regulating hair follicle development during aging, and pointed out that the massive inflammatory infiltration of aging dWAT may be a central factor hindering hair follicle regeneration (Chen et al.). Zhang *et al.* generalized the trophic and regulatory effects of the follicular sympathetic nerves and their neuropeptides in hair follicle immunity and growth (Zhang et al.).

## Wound healing and skin aging

Impaired wound healing in the elderly imposes significant pressure on clinic treatment. With aging, the skin is vulnerable to various damages due to the destruction of its barrier function and the degeneration of stem cells. Pang *et al.* revealed a crucial role of Keratin 17 in epidermal barrier repair, in which its expression is upregulated upon acute disruption of the epidermal barrier to promote lipid metabolism *via* increasing the nuclear transport of SREBP-1 and PPARγ (Pang et al.). Park *et al.* affirmed the similarities between embryonic skin development and adult skin repair processes. In this mini-review, the author summarized and compared the differences in cellular components, neighboring tissue status, and surrounding environment between the two, and provided clues for the repair of skin damage (Park).

In conclusion, the findings and ideas presented in this Research Topic provide insights into inflammation, stem cell activity, and wound healing in skin aging. With the cutting-edge experimental techniques and increasingly interdisciplinary approaches employed in this Research Topic, we have witnessed strong progress in the field. We hope that this Research Topic will pave a new way to elucidate the new mechanism of aging-related skin inflammatory diseases, hair regeneration, and wound healing, to help us advance the process of regenerative biology, and to guide the development and clinical application of anti-inflammatory and anti-aging drugs in the future.
